# Pregnancy outcome in severe OHSS patients following ascitic/plerural fluid drainage

**DOI:** 10.1186/1757-2215-7-56

**Published:** 2014-05-17

**Authors:** Jigal Haas, Yoav Yinon, Katya Meridor, Raoul Orvieto

**Affiliations:** 1 Department of Obstetrics and Gynecology, Chaim Sheba Medical Center (Tel Hashomer), Ramat Gan, Israel and Sackler Faculty of Medicine, Tel Aviv University, Tel Aviv, Israel

**Keywords:** Ovarian hyperstimulation syndrome, Singleton, Hyper-permeability, Drainage, Pregnancy outcome

## Abstract

**Background:**

Various inflammatory cytokines have been implicated in the pathophysiology of severe ovarian hyperstimulation syndrome, as well as, various pregnancy complications, including preterm labor, pregnancy induced hypertension/preeclampsia and intra-uterine growth restriction. We aim to determine whether severe OHSS, complicated by third space fluid accumulation necessitating drainage, is associated with increased risk of late obstetrics complications.

**Methods:**

We assessed the obstetrics and neonatal outcome measures of 16 patients admitted to our gynecology ward during a 6-year period, with severe OHSS complicated by third space fluid accumulation necessitating drainage.

**Results:**

Patients delivered at 37.3 ± 5.9weeks, with a mean birth weight of 3062 ± 757 gr. There was no single case of gestational diabetes, hypertensive diseases of pregnancy, nor placental abruption. Two (12.5%) patients had preterm delivery: one at 23 weeks’ gestation and one at 28 weeks’ gestation following preterm premature rupture of membrane. Another patient experienced an unexplained antepartum fetal death at 27 weeks’ gestation.

**Conclusions:**

Severe OHSS, complicated by third space fluid sequestration necessitating drainage, is not associated with adverse late pregnancy outcome, except probably for preterm labor. Following resolution of the OHSS, pregnancies should be regarded as any pregnancy resulting from IVF treatment, with special attention to prevent preterm labor.

## Background

Ovarian hyperstimulation syndrome (OHSS) is a serious complication of controlled ovarian hyperstimulation (COH), almost always presents either after human chorionic gonadotropin (hCG) administration or during early pregnancy. Its co-morbidities result from the marked ovarian enlargement and the increase in capillary permeability, with the consequent acute third-space fluid sequestration [1,2]. Moreover, despite many years of clinical experience, the pathophysiology of OHSS is poorly understood [3].

While direct action of excessive gonadotropins on ovarian tissue accounts for the ovarian enlargement, none of the hitherto reported studies have identified the cause of the third space fluid-shifting phenomenon. While the enlarged hyperstimulated ovaries are responsible for the observed increased prevalence of adnexal torsion, usually occurring during the first trimester of pregnancy [4], the most accepted hypothesis assumes that hCG induces the release of a mediator by gonadotropin-hyperstimulated ovaries, causing endothelial activation resulting in increased capillary permeability [5-7]. Several inflammatory cytokines are present in ascitic fluid of severe OHSS patients [8] and were shown to correlate with OHSS severity [9]. Moreover, inflammatory mediators, namely, C-reactive protein, leukocyte and endothelial selectins and vascular endothelial growth factor are increased after hCG administration in vivo, reflecting an inflammatory state, neutrophil and endothelial activations, respectively [6,10]. However, no significant evidence exists to prove any role of these regulators as a culprit of OHSS.

Various inflammatory cytokines, including vascular endothelial growth factor, have been implicated in the pathophysiology of several late pregnancy complications, including preterm labor [11], pregnancy induced hypertension/preeclampsia [12] and intra-uterine growth restriction [13]. Since OHSS was also related to a massive increase in systemic inflammatory cytokines that induces a systemic inflammatory response [6], a common cause/mechanism may attribute to both severe OHSS with vascular hyper-permeability and the aforementioned pregnancy complications.

The hitherto published literature regarding the relation between OHSS and pregnancy complications is limited and controversial [14-17]. This disparity in the observed outcomes probably results from the inclusion of patients with OHSS, not exclusively limited to those with significant increase in vascular permeability, as reflected by third space fluid accumulation necessitating drainage.

Prompted by the aforementioned observations, we sought to address whether severe OHSS, complicated by third space fluid accumulation necessitating drainage), is associated with increased risk of late obstetrical complications.

## Methods

We reviewed the medical files of all consecutive women admitted to our gynecology ward, during a 6 year period, due to severe OHSS following an in-vitro fertilization (IVF) treatment. Severe OHSS was defined according to Golan et al. [18] and Navot et al. [1] criteria, while early and late OHSS were defined according to Lyons et al. [19]: 3–7 days and 12–17 days following HCG ovulation triggering, respectively.

The elimination of bias in this selection, for the purposes of this study, was achieved by analyzing only those admitted with Hct level >40%, whose medical condition necessitated ascitic or pleural fluid drainage. Moreover, only those with singleton pregnancy that continued beyond the first trimester were included.

Data on patients’ demographics, infertility, obstetrical and neonatal related variables were collected from the files. Gestational age was calculated based on the day of ovum pick-up (2 weeks gestation) and was correlated to a first trimester ultrasound exam. Results are presented as mean ± standard deviation. The study was approved by the Institutional Research Ethics Board of our center.

## Results

During the study period 16 patients were hospitalized following IVF treatments due to severe OHSS necessitating ascetic or pleural fluid drainage. Mean patients’ age and body mass index were 30.8 ± 4.7 years and 21.8 ± 3.0 kg/m^2^, respectively. Causes of infertility were anovulation/ polycystic ovary syndrome in 25%, male factor infertility in 31.25% and others (unexplained, endometriosis, etc.) in 43.75% of the study group, with 10 (62.5%) of whom suffering from primary infertility. All underwent an IVF treatment with retrievals of 18 ± 5.9 oocytes and transfers of 2.2 ± 6 embryos.

Patients were admitted to our gynecology wards 14.5 + 4.7 days after HCG administration, 13 with late and 3 with early and late severe OHSS. They were hospitalized for 11.9 + 4.8 days, with a mean hematocrit level on admission of 43.8% + 2.0%.

During hospitalization, 14 underwent ascitic and 2 pleural fluid drainages. Moreover, 6 patients showed abnormally elevated liver enzymes, one suffered from oliguria, 2 had adnexal torsion and another pair suffered from thromboembolic complications (jugular and subclavian thrombosis).

Regarding the perinatal outcome, patients delivered at mean gestational age of 37.3 ± 5.9 weeks, with a mean birth weight of 3062 ± 757 gr and 1 and 5 minutes APGAR scores of 8.4 ± 2.3 and 9.3 ± 2.6, respectively. There was no single case of gestational diabetes, hypertensive diseases of pregnancy, nor placental abruption. Two (12.5%) patients had preterm delivery (<37 weeks): one at 23 weeks’ gestation and one at 28 weeks’ gestation following preterm premature rupture of membrane. A healthy baby was born, weighing 1202 gr with an APGAR score of 9/10. Another patient experienced an unexplained antepartum fetal death at 27 weeks’ gestation (with no gross fetal anomalies, normal thrombophilia evaluation and no autopsy due to parents refusal),

While 8 (50%) patients experienced a spontaneous vaginal delivery, 7 (43.75%) delivered by cesarean section and one by instrumental delivery. The indications for cesarean deliveries were either non-reassuring fetal status (n = 6), or maternal request (n = 1).

## Discussion

In the present study of patients with severe OHSS undergoing ascitic or pleural fluid drainage, we actually evaluated patients with the most severe form of OHSS, as reflected by the highest number of oocytes retrieved during their IVF treatment (18 ± 5.9 vs 13 ± 5.2, 13.7 ± 5.2 and 16.8 ± 5.9 oocytes) and the longest hospitalization period (11.9 ± 4.8 vs 10.2 ± 7.2 and 4.5 ± 2.9 and 6.6 ± 4.71 days), as compared to the hitherto published studies [16,15,17], respectively. Moreover, a closer look at the aforementioned studies (Table 1) reveals that none of them related exclusively to patients who underwent ascitic or pleural fluid drainage, with no mention on the prevalence of those (with singleton pregnancy) undergoing drainage [15-17]. Moreover, two studies did not differentiate between singleton and twin pregnancies [14,16].

**Table 1:** Summary of the hitherto published studies relating to pregnancy complications in patients with severe OHSS

	**Abramov et al.**[14]	**Wiser et al.**[15]	**Courbiere et al.**[16]	**Haas et al.**[17]	**Present study**
All pregnancies				125	
Number of pregnancies	68	165	40		
Gestational age at delivery (weeks)	35.8 ± 3.0		37 ± 1.5		
Birth weight (grams)	2221 ± 252				
Cesarean Section rate (%)	44.1				
Preterm labor < 37 wks (%)	44		36		
Hypertensive diseases of pregnancy (%)	13.2		21.2		
IUGR (%)	1.5				
Gestational Diabetes (%)	5.9		6		
IUFD (%)	1.5				
Adnexal torsion (%)			5	4.8	
Thromboembolism (%)	1.5		10	1.6	
Paracentesis (%)			2.5	32.8	
Singleton pregnancies					
Number of pregnancies	29	101	31	78	16
Gestational age at delivery (weeks)	37.2 ± 3.2	39 ± 1.9		37.9 ± 3.3	
Birth weight (grams)	2785 ± 356	3045 ± 544	3032 ± 545	2854 ± 610	37.3 ± 5.9
Cesarean Section rate (%)	24.1	24		28.2	43.75
Preterm labor < 37 wks (%)	28	1		20.5	12.5
Hypertensive diseases of pregnancy (%)		6.9		8.9	0
IUGR (%)				2.6	
Gestational Diabetes (%)		9.9		17.9	0
IUFD (%)				1.2	6.2
Adnexal torstion (%)					12.5
Thromboembolism (%)					6.2
Paracentesis (%)					100

While our study group of patients with the most severe form of OHSS demonstrated all the well-documented complications, namely, elevated liver enzymes, oliguria, adnexal torsion and thromboembolism, no single case of gestational diabetes, hypertensive diseases of pregnancy, or placental abruption was observed.

The findings reported herein, therefore, indicate that singleton pregnancies complicated by severe OHSS, necessitating ascitic or pleural fluid drainage, are not at risk for obstetric complications, except probably for preterm labor. While, studies relating to hypertensive diseases of pregnancy, gestational diabetes and IUGR show contradictory results [14-17], the observed increased prevalence of preterm labor (12.5%) in our study, is in accordance with previous studies [14,16,17], and is also a well-known complication of IVF pregnancies in general [20]. Moreover, the observed single case of unexplained antepartum fetal death requires attention and raises the question whether it is accidental or is associated to severe OHSS.

Despite the small sample size and the inherent disadvantages of a retrospective study design, we would expect a significantly higher prevalence of these obstetric complications among this subgroup of severe OHSS patients with ”endothelial” activation, as reflected by the third space fluid sequestration, necessitating drainage. The observed lack of association between severe OHSS complicated with third space fluid sequestration and an increased prevalence of the aforementioned obstetric complications, except probably for preterm labor, actually excludes the possibility of a common mechanism responsible for these abnormalities.

## Conclusions

To conclude, we have demonstrated that severe OHSS, complicated by third space fluid sequestration necessitating drainage, is not associated with adverse late pregnancy outcome (i.e. intrauterine growth restriction and pregnancy induced hypertension/preeclampsia), except for preterm labor. Following resolution of the OHSS, pregnancies should be regarded as any pregnancy resulting from IVF treatment, with special attention to identify and treat preterm birth. Further large prospective studies are required in order to determine whether severe OHSS complicated with third space fluid sequestration, is associated with late pregnancy complications or whether there are preexisting factors predisposing women to develop both OHSS and various pregnancy complications.

## Abbreviations

COH: Controlled ovarian hyperstimulation; hCG: Human chorionic gonadotropin; IVF: In vitro fertilization; OHSS: Ovarian hyperstimulation syndrome.

## Competing interests

The authors declare that they have no competing interests.

## Authors’ contributions

JH- Collected the data, performed the statistical evaluations, assisted in writing the paper and edited it in all its revisions. YY- Collected the data and edited the paper in all its revisions. KM- Collected the data and edited the paper in all its revisions. RO- The principal investigator, designed the study, analyzed the data, assisted in writing the paper and edited it in all its revisions. All authors read and approved the final manuscript.
